# *De Novo* Genome Assembly of *Populus simonii* Further Supports That *Populus simonii* and *Populus trichocarpa* Belong to Different Sections

**DOI:** 10.1534/g3.119.400913

**Published:** 2019-12-06

**Authors:** Hainan Wu, Dan Yao, Yuhua Chen, Wenguo Yang, Wei Zhao, Hua Gao, Chunfa Tong

**Affiliations:** Key Laboratory of Forest Genetics & Biotechnology of Ministry of Education, Co-Innovation Center for Sustainable Forestry in Southern China, Nanjing Forestry University, Nanjing 210037, China

**Keywords:** genome assembly, PacBio sequencing, Illumina sequencing, genetic linkage maps, *Populus simonii*

## Abstract

*Populus simonii* is an important tree in the genus *Populus*, widely distributed in the Northern Hemisphere and having a long cultivation history. Although this species has ecologically and economically important values, its genome sequence is currently not available, hindering the development of new varieties with wider adaptive and commercial traits. Here, we report a chromosome-level genome assembly of *P. simonii* using PacBio long-read sequencing data aided by Illumina paired-end reads and related genetic linkage maps. The assembly is 441.38 Mb in length and contain 686 contigs with a contig N50 of 1.94 Mb. With the linkage maps, 336 contigs were successfully anchored into 19 pseudochromosomes, accounting for 90.2% of the assembled genome size. Genomic integrity assessment showed that 1,347 (97.9%) of the 1,375 genes conserved among all embryophytes can be found in the *P. simonii* assembly. Genomic repeat analysis revealed that 41.47% of the *P. simonii* genome is composed of repetitive elements, of which 40.17% contained interspersed repeats. A total of 45,459 genes were predicted from the *P. simonii* genome sequence and 39,833 (87.6%) of the genes were annotated with one or more related functions. Phylogenetic analysis indicated that *P. simonii* and *Populus trichocarpa* should be placed in different sections, contrary to the previous classification according to morphology. The genome assembly not only provides an important genetic resource for the comparative and functional genomics of different *Populus* species, but also furnishes one of the closest reference sequences for identifying genomic variants in an F_1_ hybrid population derived by crossing *P. simonii* with other *Populus* species.

*Populus simonii* (Salicaceae: *Populus*) is one of the most important native trees in northern China, which is mainly distributed from Qinghai to the east coast and from the Heilongjiang River to the Yangtze River (Wei *et al.* 2013). It is a primary tree species in the three northern regions of China (northwest, north, and northeast) and plays an important role in preventing desertification, reducing soil erosion, counteracting wind damage, and fixing sand dunes. Because of its drought resistance, barren tolerance, wide adaptability, strong rooting ability and interspecific cross-compatibility, *P. simonii* has been regarded by poplar breeders as one of the best parents for breeding poplar clone varieties (Zhu *et al.* 2018). Based on this recognition, we established an F_1_ hybrid population by crossing *P. deltoides* and *P. simonii* and used this population to construct genetic linkage maps and map quantitative trait loci (QTL) (Tong *et al.* 2016; Mousavi *et al.* 2016).

The acquisition of genomic sequences provides a solid basis for understanding the biological significance of individual species. In our previous study, paired-end (PE) reads from the Illumina Hiseq platform were used to construct a draft genome sequence of *P. simonii* as a reference sequence for generating single nucleotide polymorphism (SNP) markers that can be used to construct genetic linkage maps, but the assembly has relatively poor continuity and lacks annotated information on repetitive sequences and functional genes (Mousavi *et al.* 2016). In studies of the stress resistance of *P. simonii* (Chen *et al.* 2013; Song *et al.* 2013), some transcriptional regulators related to drought and cold tolerance were found by analyzing transcriptome sequencing data, but the lack of genomic information for *P. simonii* made it difficult to further study the molecular mechanism of stress resistance. Although the genome assemblies of *Populus deltoides* (http://www.phytozome.net), *Populus euphratica* (Ma *et al.* 2013), and *Populus trichocarpa* (Tuskan *et al.* 2006) are available online, there are significant differences in the genomes of different poplar species (Yang *et al.* 2017; Liu *et al.* 2017; Ma *et al.* 2019). Moreover, comparative genomics based on *de novo* assembly in poplar is still in its infancy. Further development of genomic resources for *P. simonii* will provide unique opportunities for comparative genomics and will also accelerate understanding of the evolutionary process underlying the phenotypic and adaptive differences exhibited by *P. simonii*. To improve the understanding of the *P. simonii* genome, explore its complex stress-resistance characteristics, and further study its genome through genetic linkage analysis and QTL mapping, it is necessary to obtain a genome assembly of *P. simonii* that possesses high continuity.

To date, more than 200 plant genomes have been sequenced (Vanburen *et al.* 2018), including some highly heterozygous plant genomes such as Norway spruce (Nystedt *et al.* 2013) and *Liriodendron* (Chen *et al.* 2019). Recently, some *de novo* assemblies of plant genomes such as *Ganoderma lucidum* (Liu *et al.* 2012), *Siraitia grosvenorii* (Itkin *et al.* 2016), and *Populus pruinosa* (Yang *et al.* 2017) were obtained using next-genenation sequencing (NGS) technology. Due to the complexity of plant genomes, the draft assembled sequences obtained with NGS platforms generally have poor continuity, and many gaps remain, resulting in the potential loss of a large number of important biological characteristics (Vanburen *et al.* 2018). With recent advances in sequencing technology, several new platforms such as PacBio and Oxford Nanopore have been used to assemble large and complex genomes from tens of thousands of long individual reads. Several of these genomes, including those of opium poppy (Guo *et al.* 2018), *Panicum miliaceum* (Shi *et al.* 2019), maize (Yang *et al.* 2019), and strawberry (Edger *et al.* 2019), were assembled using reads generated on the PacBio sequencing platform, while others such as those of *Arabidopsis thaliana* (Michael *et al.* 2018), *Chrysanthemum nankingense* (Song *et al.* 2018) and *Tectona grandis* (Yasodha *et al.* 2018) were assembled using the Nanopore sequencing technology.

In this study, we report a high-quality genome sequence of *P. simonii* that was mainly assembled using long reads generated by the PacBio Sequel system. These reads were incorporated with paired-end (PE) short reads from the Illumina HiSeq platform and with high-density genetic maps of *P. deltoides* and *P. simonii*. The draft genome sequence is of high quality in terms of both genome integrity and base quality. Genomic repetitive sequences were analyzed, and a large number of protein-coding genes were predicted and annotated. Concomitant phylogenetic analysis provided genome-level evidence for the placement of *P. simonii* and *P. trichocarpa* in different sections. The genome assembly of *P. simonii* provides a valuable resource for identifying genome variants among individuals, performing comparative genomics within and between species, and mining candidate genes that underlie ecologically and economically important traits.

## Materials and Methods

### Sampling and sequencing

As the male parent in an F_1_ hybrid population, a single *P. simonii* tree was chosen from a forestland managed by the Luoning Forest Bureau of Henan Province, China and planted at the Xiashu Forest Farm of Nanjing Forestry University, Jurong, Jingsu Province, China (Mousavi *et al.* 2016). Fresh leaves were collected from the paternal tree, immediately transferred to liquid nitrogen, and stored until DNA extraction. Genomic DNA was extracted using the CTAB protocol (Porebski *et al.* 1997), and a library with an insert size of 20 kb was constructed using a BluePippin DNA size selection instrument (Sage Science, MA, USA) with a lower size limit of 10 kb. The prepared library was sequenced using P6/C4 chemistry according to the manufacturer’s protocols (Pacific Biosciences, USA). Single-molecule real-time (SMRT) sequencing of long reads was performed at BMK (Biomarker Technologies Corporation, Beijing, China) and at FTC (Frasergen Technologies Corporation, Wuhan, China) on the PacBio Sequel platform (Pacific Biosciences, USA). After the removal of adapter sequences using SMRTlink (v7.0.1; https://www.pacb.com/support/software-downloads), the remaining sequences were used for genome assembly and error correction.

In addition, using the same individual tree described above, we sequenced the whole genome of *P. simonii* by the Illumina HiSeq 2000 sequencing platform at BMK. The detailed procedure used for whole-genome sequencing is described in Mousavi *et al.* (2016). The PE reads data are available from the NCBI SRA database under the accession number SRP071167. These whole-genome sequencing data were filtered using the NGC QC toolkit with default parameters (Patel and Mukesh 2012) to obtain high-quality data for correcting the contigs assembled with the PacBio sequencing data.

For the purpose of genome annotation, we also performed RNA sequencing of fresh leaf tissue isolated using TRIzol Reagent (Invitrogen, USA). The RNA libraries were prepared using the TruSeq RNA Library Preparation Kit (Illumina, CA, USA), and PE sequencing with a read length of 90 bp was then conducted on the Illumina HiSeq 2000 platform at Beijing Genomics Institute (BGI). These transcriptome reads were already deposited in the SRA database at the NCBI with the accession number SRR9113443. The RNA sequencing data were also filtered using the NGC QC toolkit with default parameters (Patel and Mukesh 2012).

### Genome assembly and correction

The genome size of *P. simonii* was estimated based on the k-mer method using the sequencing data from the Illumina DNA library (Lamichhaney *et al.* 2016). High-quality reads were subjected to 17-mer distribution using the Jellyfish program (Marçais and Kingsford 2011). The genome size was estimated according to the formula G=(Ktotal-Kerror)/D, where Ktotal is the total number of K-mers, Kerror is the number of K-mers at low frequency (frequency <=1), D is the depth of K-mer, and G is the final predicted genome size. In addition, the genome heterozygosity was estimated using GenomeScope software (Vurture *et al.* 2017).

**Figure 1:**
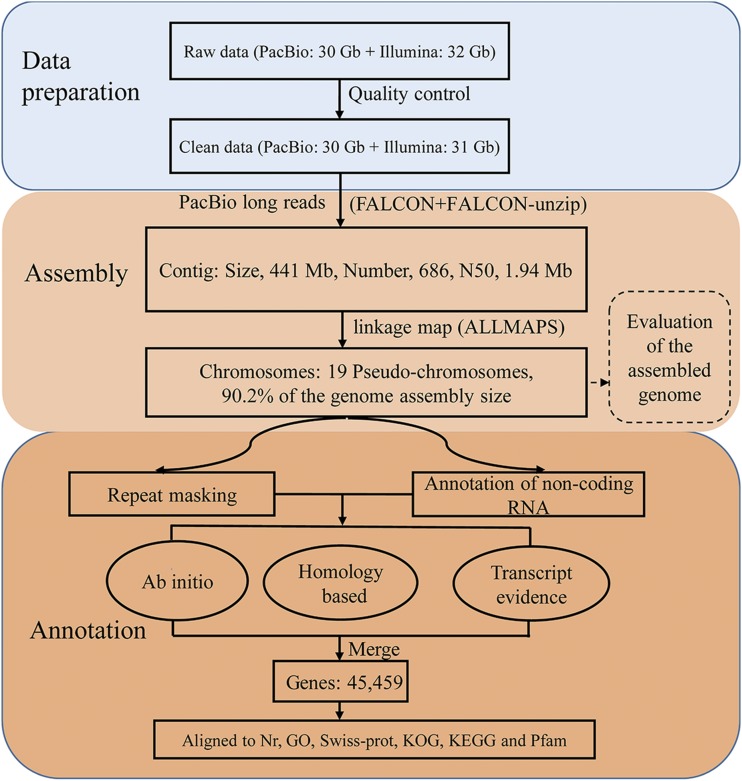
Integrated work-flow for sequencing, assembly and annotation of the *Populus simonii* genome.

As shown in Figure 1, we first obtained the contigs of *P. simonii* by assembling the long reads sequenced from the PacBio Sequel platform. The reads data in BAM format were transformed into FASTA format by the standard PacBio SMRTlink software package after removal of adapter sequences. After further filtering out short sequences (<500 bp), the remaining reads were used in the subsequent analysis. The contig assembly of *P. simonii* was conducted using the FALCON assembler (v0.3.1) (Chin *et al.* 2016). FALCON is a hierarchical genome assembly software package developed by PacBio that uses the following steps to generate a genome assembly from a set of sequencing reads: (1) Raw subreads overlapping for error correction; (2) Preassembly and error correction; (3) Overlapping detection of the error-corrected reads; (4) Overlap filtering; (5) Construction of a graph from the overlaps; and (6) Construction of contigs from the graph. Through adjusting the parameters of the FALCON software in error correction and assembly several times, the following best parameters for assembly were obtained: length_cutoff = 1000; length_cutoff_pr = 1000; pa_daligner_option = -e.70 -l1000 -k18 -h480 -w8 -s100; falcon_sense_option =–output-multi–min-cov-aln 4–min-idt 0.70–min-cov 4–max-n-read 200; overlap_filtering_setting =–max-diff 60–max-cov 100–min-cov 2. Due to the complexity of forest genomes (Minio *et al.* 2019), we further used FALCON-unzip with the default parameters to phase the reads based on heterozygous SNPs identified in the initial assembly. The purpose of this step was to produce a set of partially phased primary contigs and fully phased haplotigs (Chin *et al.* 2016).

To improve the accuracy of the genome assembly, we polished the contigs described above in two ways. First, all the SMRT clean reads were aligned to the contigs of *P. simonii* using the command *blasr* in SMRTlink; then, Arrow software (Chin *et al.* 2013) was used to correct the errors in the contigs. Next, second-round correction was performed using the high-quality Illumina short reads. The PE reads data were aligned to the contigs using the command *mem* in the software BWA (v0.7.15) (Li and Durbin 2009); SAMtools software (v1.8) (Li *et al.* 2009) was then used to convert the output files to BAM format. We applied the software Pilon (v1.22) (Walker *et al.* 2014) to the alignment results to further correct the contigs of the assembly under the default parameters.

### Assembly integration with genetic linkage maps

Two parental linkage maps of *P. deltoides* and *P. simonii* were used to anchor the contigs into chromosomes using the software ALLMAPS (Tang *et al.* 2015). The linkage maps, which were constructed in a previous study (Yao *et al.* 2018), contained a total of 3,913 SNPs on the maternal map of *P deltoides* and 2,584 SNPs on the paternal map of *P. somonii*. To match each linkage group with the chromosome in *Populus*, we mapped a 41-bp sequence flanking each SNP to the reference genome of *P. trichocarpa* using the program *blastn* from NCBI (ftp://ftp.ncbi.nlm.nih.gov/blast/executables/blast+/2.8.1/). If the majority of SNPs in a linkage group were mapped to a chromosome, the linkage group was considered to correspond to the chromosome. The sequence representing a SNP was also aligned to the contigs assembled above by setting the query coverage equal to 90%. After that, two csv-formatted files were prepared for each parental linkage map according to the requirement of ALLMAPS; these files contained records for each mapped SNP, including the contig identifier, the position on the contig, the linkage group identifier, and the position in the linkage group. Finally, the anchoring process was conducted using ALLMAPs with equal weights for the two linkage maps.

### Assessment of the genome assembly

To evaluate the base level accuracy of the genome assembly, high-quality PE reads data were mapped to the assembled genome using BWA software to generate mapping ratio statistics. Then, the erroneous bases and homozygous SNP loci in the genome were identified using the software Freebayes (v1.2.0) (Garrison and Marth 2012) with the BAM files generated in BWA. We further evaluated the completeness of the genome assembly using Benchmarking Universal Single-Copy Orthologs software (BUSCO v3) (Simão *et al.* 2015) with the embryophyta_odb10 database (http://busco.ezlab.org) and the parameter settings “-m genome -c 30”. The completeness of genome assembly was assessed on the basis of the proportion of the complete BUSCOs present in the genome.

### Repetitive sequences and noncoding RNA annotation

Plant genomes, especially complex plant genomes in which many of the repetitive sequences are transposable elements (TEs), usually contain large numbers of repetitive sequences (Xu *et al.* 2019). To identify transposable elements, the software RepeatModeler (Tarailo-Graovac and Chen 2009) was used to identify *de novo* repeat types in *P. siomonii*. Then, combination of the results from RepeatModeler with the repeat sequence database Repbase (Bao *et al.* 2015) was used as the final repeat sequence library for subsequent analysis with RepeatMasker (Tarailo-Graovac and Chen 2009). This step yields a more accurate annotation result for repetitive elements in *P. simonii*.

In the process of annotating noncoding RNAs (ncRNA), we identified miRNA and snRNA genes in the genome of *P. simonii* by searching the Rfam database using INFERNAL software with default parameters (v1.1.2) (Kalvari *et al.* 2018). Moreover, we used tRNAscan-SE software with default parameters for tRNA annotation (v1.3.1) (Lowe and Eddy 1997) and RNAmmer for rRNA annotation (v1.2) (Lagesen *et al.* 2007).

### Gene prediction

We identified the protein-coding genes of *P. simonii* using *ab initio* prediction, homology-based prediction, and RNA-seq-assisted prediction methods. The transcripts were obtained by *de novo* assembling the RNA-seq data of *P. simonii* with Trinity (v2.4.0) (Haas *et al.* 2013). Before gene prediction, an initial gene model file was constructed by analyzing the transcriptomic data with the software PASA (v2.3.3) (Campbell *et al.* 2006). In addition, the repetitive sequences of the transcripts were screened according to the annotations of repetitive sequences to prevent them from interfering with the results of gene prediction. Next, the Augustus (v3.3.1) (Stanke *et al.* 2006), SNAP (v2006-07-28) (Korf 2004) and GeneMark (v4.46) (Ter-Hovhannisyan *et al.* 2008) software packages were applied with default parameters to create three *ab initio* training files incorporating the use of PASA for gene model training. For homology-based prediction, protein sequences from 4 closely related species, *Salix suchowensis* (Dai *et al.* 2014), *Populus trichocarpa* (Tuskan *et al.* 2006), *Populus deltoides* (https://phytozome.jgi.doe.gov/pz/portal.html), and *Populus euphratica* (Ma *et al.* 2013), were aligned to the genome assembly of *P. simonii* using tblastn with the default parameters. Furthermore, Exonerate software (Slater and Birney 2005) was used to polish the blast hits to obtain exact intron and exon positions. Finally, all the predictions results were integrated using the software package MAKER (v3.01.02) (Holt 2011) to generate a consensus gene set, and the quality of the gene annotation was assessed using BUSCO (v3) with the parameters set as follows: “-c 30 –m proteins –l embryophyta_odb10”.

### Functional annotation of protein-coding genes

Functional annotations of the protein-coding genes in *P. simonii* were obtained according to the best matches by aligning the predicted protein sequences to well-known databases using the local blastp command with an e-value threshold of 1e-5. The databases included Nonredundant protein sequences (Nr) as of March 12, 2019, eukaryotic orthologous groups of proteins (KOG) as of September 9, 2018 (Tatusov *et al.* 2001), the Kyoto Encyclopedia of Genes and Genomes (KEGG) as of July 1, 2019 (Ogata *et al.* 2000) and Swissprot as of August 10, 2019 (Bairoch and Boeckmann 1991). To derive the annotation information for motifs and domains, we searched the protein sequences in the Pfam database (Pfam 28) as of May 20, 2015 (Zdobnov and Apweiler 2001) using the software hmmer (v3.1b2) (Mistry *et al.* 2013) with default parameters. The blast hits of the protein-coding genes were mapped to the Gene Ontology (GO) terms using the BLAST2GO pipeline (Conesa *et al.* 2005). InteractiVenn software (Heberle *et al.* 2015) was used to plot a Venn diagram of the number of protein-coding genes with functional annotations based on the six databases described above.

### Comparative and evolutionary genomics

To cluster families from protein-coding genes, we downloaded the online protein data for eight other species, including three species in the genus *Populus*. We retrieved protein data of *P. trichocarpa* (v3.1), *P. deltoides* WV94 (v2.1), *Ricinus communis* (v0.1), *Oryza sativa* (v7), *Carica papaya* ASGPB (v0.4), *Manihot esculenta* (v6.1), and *Salix purpurea* (v1.0) from Phytozome (https://phytozome.jgi.doe.gov/pz/portal.html). The data for *P. euphratica* (v1.0) were downloaded from NCBI (https://www.ncbi.nlm.nih.gov/genome/). The *Salix suchowensis* (v4.1) data were obtained from the ftp site at Nanjing Forestry University (ftp://plantgenie.org/Data/PopGenIE/Salix-suchowensis). To remove redundant sequences caused by alternative splicing variations, we retained only the gene model at each gene locus that encodes the longest potential protein sequence. We then employed DIAMOND software to identify potentially orthologous gene families among the filtered protein sequences in these species using a maximum e-value of 1e-5 (Buchfink *et al.* 2015). The pipeline OrthoFinder was used to cluster gene families based on the aligned results under the default parameters (v2.3.3) (Emms and Kelly 2015). STRIDE software (Emms and Kelly 2017) was used to identify gene duplication events based on the orthgroups generated by OrthoFinder.

The *P. simonii* genome was compared to the *P. trichocarpa* genome (v3.1) using the MUMmer software package (v3.23) (Kurtz *et al.* 2004). The chromosome-level sequences of *P. simonii* were aligned to the reference genome of *P. trichocarpa* with the PROmer script in the package followed a filtering process using the delta-filter script with parameter setting as “-i 100 -l 150”. A postscript file was then generated using the mummerplot script for visualization of the genomic structure comparison.

To reveal the phylogenetic relationship between *P. simonii* and other *Populus* species, we conducted phylogenetic analysis using the orthologs from the single-copy gene families. MAFFT software (v7.158b) (Katoh and Standley 2013) was used to generate multiple sequence alignments of protein-coding sequences for each single-copy gene with an accurate option (LINS-i). The software trimAl (v1.2) (Capella-Gutiérrez *et al.* 2009) was used with the default parameters to remove poorly aligned positions and divergent regions. The alignments for all the single-copy genes were then concatenated to form a super matrix. The super matrix was used for phylogenetic tree construction by RAxML software (v8.2.12) (Stamatakis 2014) with the model JTT+G+F (determined by the BIC model selection criterion) and bootstrap replicates of 1000. Next, r8s software (v1.71) (Sanderson 2003) was applied to estimate the divergence time among species with selected parameter settings as follows: “blformat lengths=persite nsites=462057 ulrametric=no; set smoothing=100; divtime method=PL algorithm=TN” and the others as defaults. The divergence times of *Oryza sativa* (115-308 Mya), *Ricinus communis* (70-86 Mya), *and Salix purpurea* (12-48 Mya) obtained from the TimeTree database (http://www.timetree.org/) were used as the calibration points. Finally, the phylogenetic tree was visualized in FigTree (v1.4.3) (Drummond and Rambaut 2007).

To investigate whole-genome duplication (WGD) events in *P. simonii*, the distribution of synonymous substitution rate (Ks) was obtained from its CDS sequences, compared with *P. trichocarpa*. The pipline wgd (Zwaenepoel and Van de Peer 2019) was used to calculate the Ks distributions from paralogous sequences in each of the two species. We also used CAFÉ software (De Bie *et al.* 2006) to analyze gene family evolution according to the phylogenetic relationships and divergence times, setting the parameters as “load –p 0.05 –t 20; lambda –s”.

### Data availability

The data of the whole-genome PE short reads from the Illumina platform, the two batches of long reads from the PacBio system, and the transcriptome reads have been deposited in the SRA database at the National Center for Biotechnology Information (NCBI) with accession numbers of SRP071167, SRR9112943, SRR9887262, and SRR9113443, respectively. The genome assembly of *P. simonii* is available under the GenBank assembly accession number GCA_007827005.2. Statistical information on the PacBio Sequel raw data can be found in File S1. The repetitive sequence annotation was saved in File S2 in fasta format. Annotation of noncoding RNAs can be found in File S3. The protein sequences with the predicted genes are contained in File S4 in fasta format. The genome annotation was saved in File S5 in gff3 format. Gene functional annotation information can be found in File S6. The supplementary tables (Tables S1-S7) and figures (Figures S1-S11) can be found in File S7. Supplemental material available at figshare: https://doi.org/10.25387/g3.9905492.

## Results

### Sequencing

SMRT sequencing of long reads was conducted on a PacBio Sequel platform with 6 cells. After filtering out the low quality and adapter sequences, a total of 3,412,303 reads were obtained (Table 1 and Figure S1). After further filtering out the short sequences (<500 bp), 3,147,743 high-quality reads up to 29.65 Gb remained, with an average read length of 9,418 bp and a longest read length of 112,390 bp (Table 2 and File S1).

**Table 1:** Clean data*#**^a^* generated by the PacBio sequel platform

Cell ID#*^b^*	Reads Num#*^c^*	Total Bases(bp)	Reads N50(bp)	Mean Length(bp)	Longest Read(bp)
4_D03	427,147	2,583,955,526	11,500	6,049	70,237
3_G01	651,595	4,705,217,582	12,948	7,221	84,525
4_H01	329,754	2,855,303,758	13,761	8,659	61,755
2_B05	509,634	5,109,633,303	14,523	10,026	84,905
1_A09	827,878	6,622,523,144	13,683	7,999	96,519
G01	666,295	7,840,437,521	19,479	11,767	112,390
Total	3,412,303	29,717,070,834	15,473	8,709	112,390

a Clean data: The sequences remaining after filtering out low-quality reads and adapters.

b Cell ID: Chip ID.

c Reads Num: number of reads.

**Table 2:** Statistics of high-quality reads data and library information

Data Type	Platform	Number of Reads	Bases(bp)	Insert Size(bp)	Average Read Length(bp)
DNA Sequence (short insert)	Illumina	256,124,774	25,868,602,174	300-500	101
DNA Sequence (long insert)	PacBio Sequel	3,147,743	29,647,972,079	20,000	9,418
RNA-Seq (short insert)	Illumina	94,464,940	8,501,844,600	300-500	90

The short fragments of the DNA and RNA libraries were sequenced using the Illumina HiSeq 2000 sequencing platform. A total of 326,922,690 PE reads representing 32.02 Gb were obtained by genomic sequencing. After filtering out the low-quality sequences, 256,124,774 high-quality reads representing 25.87 Gb of genomic data remained. For the transcriptome sequencing, we obtained approximately 8.50 Gb of RNA-Seq data after filtering out the low-quality reads (Table 2).

### Genome size estimation, assembly and correction

To determine the amount of data needed for sequencing, we estimated the genome size of *P. simonii*. The genome size was predicted based on K-mer analysis using the software Jellyfish with the Illumina genomic sequencing data (Marçais and Kingsford 2011). Taking the k-mer size as 17 bp, a total of 21,770,605,790 k-mers were generated with 932,308 error kmers and a kmer depth of 53, leading to an estimated genome size of approximately 411 Mb and an estimated heterozygosity rate of 1.34% (Figure S2).

*De novo* assembly of the *P. simonii* genome was performed using FALCON software with the PacBio long reads data. To improve the accuracy of the PacBio data, we first used the self-correcting program of FALCON to correct the HQ long reads, obtaining 1,690,300 reads up to 16.15 Gb (∼34x coverage) with an average length of 9,553 bp for *de novo* assembly. After performing the primary assembly, we obtained a draft genome size (G1) of 447 Mb with 911 contigs and a contig N50 of 1.89 Mb (Table 3). We then used FALCON-unzip to phase the primary contigs, obtaining 440 Mb partially phased primary contigs (G2) and 328 Mb fully phased haplotigs with contig N50s of 1.93 Mb and 202 kb, respectively (Table 3). Next, the assembled genome was error-corrected using Arrow software with the PacBio data to obtain a consensus sequence. This step corrected 946,062 insertions, 142,410 deletions, and 540,656 substitutions, leading to a corrected genome size of 441 Mb. To further improve the accuracy of the genome assembly, we also used Pilon software and the Illumina genomic reads to correct the consensus sequences again. This identified 181,316 insertion sites, 76,138 deletion sites, and 152,077 substitution sites that required correction. After the two rounds of error correction, the final genome assembly (G3) of *P. simonii* contained 686 contigs and had a total length of 441 Mb and a contig N50 of 1.94 Mb; the longest contig was equal to 8.12 Mb, and the GC content was 33.65% (Table 3).

**Table 3:** Statistics of the *Populus simonii* assembly

Method#*^a^*	Type	Genome Size (Mb)	Sequence number	Longest sequence (Mb)	N50 length (Mb)
FALCON (G1)	contig	447	911	8.12	1.89
FALCON-unzip (G2)	contig	440	722	8.13	1.93
G3	contig	441	686	8.12	1.94
ALLMAPS (G4)	scaffold	441	369	52.00	19.6

a G1: assembled by the software FALCON; G2: assembled by the software FALCON-unzip; G3: corrected by the software Pilon and Arrow; G4: assembled by combining the genetic maps using the software ALLMAPS.

### Integration of the genome assembly using linkage maps

The two high-quality genetic linkage maps from our previous study (Yao *et al.* 2018) were used to anchor the contigs assembled above into chromosomes with genetic and genomic distances. Those contigs, each containing at least one marker on the linkage maps, were assigned to 19 chromosomes. The maternal and paternal genetic maps were integrated to form a consensus map using ALLMAPS. The resulting consensus map consisted of 5,971 unique markers, equivalent to an average physical marker density of 15.0 markers/Mb. Of 686 contigs in the draft genome assembly of *P. simonii*, 336 were aligned on the genetic maps by at least one marker, accounting for 90.2% of the assembled genome size (398 Mb of the 441 Mb assembly). In addition, 225 contigs (representing 75.9% of the anchored contigs and comprising 84.1% of the total genome length) were anchored with two or more markers so that their orientations on the chromosomes could be determined. Due to lack of markers or location conflicts, 350 contigs and 126 markers were not anchored to chromosomes; these represented only 9.8% of the total genome length (Figure S3 and Table 4). The adjacent contigs in each chromosome were filled with 100 ‘N’s. In summary, the final assembled genome of *P. simonii* contained 19 chromosome sequences and 350 unplaced contigs and had a total length of 441 Mb and a sequence N50 of 19.6 Mb (G4).

**Table 4:** Summary of the consensus map

	Anchored	Oriented	Unplaced
Markers (unique)	5,971	5,755	126
Markers per Mb	15.0	15.5	2.9
N50 contigs#*^a^*	70	69	0
Total number of contigs	336	255	350
Contigs with 1 marker	44	0	44
Contigs with 2 markers	32	13	24
Contigs with 3 markers	26	21	7
Contigs with >=4 markers	234	221	3
Total bases (bp)	398,322,698	371,036,734	43,052,653
Percentage of genome	90.2%	84.1%	9.8%

a N50 contigs: the number of contigs longer than or equal to the contig N50.

### Assembly evaluation

To evaluate the accuracy of our genome assembly, the 25.87 Gb Illumina genomic DNA sequencing data were aligned to the final assembled genome of *P. simonii* (G4) using BWA. The results showed that 97.56% of the reads were mapped to the genome assembly, indicating a high mapping ratio. We then used the software Freebayes to identify the erroneous bases in the *P. simonii* genome assembly. A total of 80,463 homozygous mutation sites were identified with a mutation rate of 1.82×10^−4^, indicating a high-quality genome assembly.

We also evaluated the completeness of the genome assembly (G3, Table 3) using BUSCO with the embryophyta_oda10 database. The results showed that 97.9% of the complete BUSCO genes were identified in the assembly (1,347 *vs.* 1,375 genes), with 0.5% partial BUSCOs identified and only 1.6% missed (Table S1). Overall, all the results suggest that the quality of the assembly is high with respect to the base level accuracy and the completeness of the assembly.

### Repetitive sequence and noncoding RNA analysis

Repetitive sequence analysis of the assembly (G3, Table 3) showed that the repetitive sequences comprised approximately 183 Mb (41.47%) of the *P. simonii* genome (File S2); of these, interspersed repeats accounted for 40.17%, simple repeats occupied approximately 5,723,970 bp (1.30%), and DNA elements made up 3.99% (Table 5). In the noncoding RNA analysis, we identified 1,153 miRNAs, 1,177 tRNAs, 290 rRNAs, and 618 snRNAs in the *P. simonii* genome (Table S2; File S3).

**Table 5:** Summary statistics of annotated repeats

Type	Number of elements	Length occupied (bp)	Percentage of sequence
DNA	40,251	17,621,301	3.99
LINE	4,300	3,138,076	0.71
SINE	8,971	1,758,144	0.40
LTR	69,002	47,295,184	10.72
Unknown	232,651	107,493,878	24.35
Simple repeats	143,553	5,723,970	1.30
Total	498,728	183,030,553	41.47

### Gene prediction and functional annotation

We predicted genes with *ab initio*, homologous annotation and transcriptome sequencing-based methods using the MAKER software package. A total of 45,459 protein-coding genes were predicted in the *P. simonii* genome; they were distributed in 369 scaffolds (G4, Table 3) with an average of 123 genes per scaffold (Table S3; File S4). BUSCO analysis showed that 94.9% of the complete BUSCO genes were identified in the protein-coding genes (Table S1). All gene structure information for the genome is reported in File S5.

**Figure 2:**
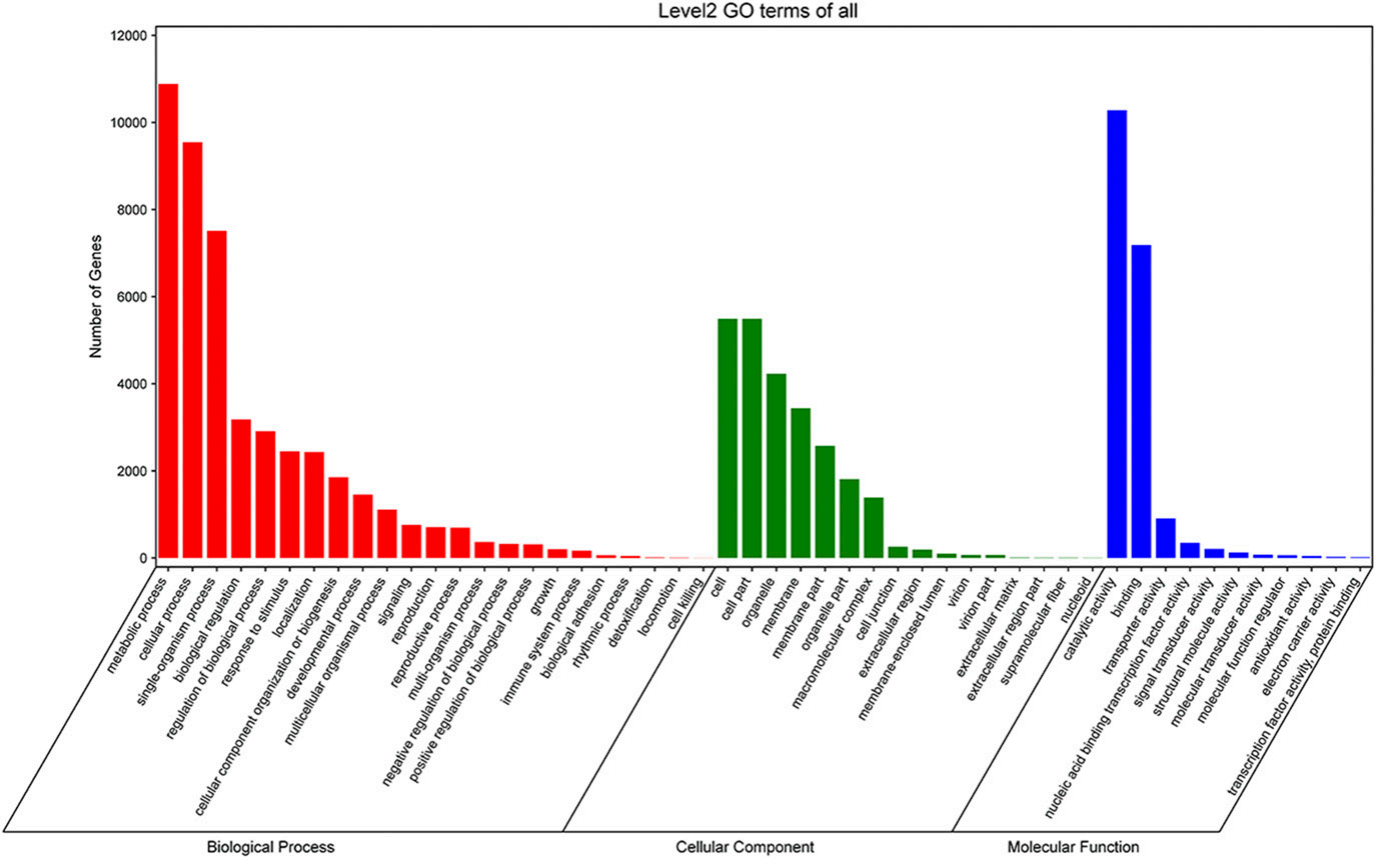
Gene Ontology (GO) function annotation of *Populus simonii* using WEGO 2.0 (Ye *et al.* 2018). The horizontal axis shows the GO classification types, and the vertical axis represents the number of annotated protein-coding genes.

To obtain motif, domain, pathway and other functional information on the predicted genes, the predicted protein sequences were searched in the publicly available databases, and the annotated results in each database were counted. By aligning to the KOG database, 10,257 proteins were assigned to 25 KOG categories; of these, the T function classification (Signal transduction mechanisms) contained approximately 1,538 predicted genes, and the R function classification (general function prediction only) contained 1,493 predicted genes (Figure S4). Moreover, 17,860 genes were assigned to corresponding GO terms, accounting for 39.3% of the total number of predicted proteins. The GO annotation classification includes three main categories: “biological process”, “cellular component” and “molecular function”. In the molecular function category, “catalytic activity” was the largest subcategory, followed by the “binding” subcategory. In the cellular component category, “cell” and “cell part” were the two main subcategories. In the biological process category, the majority of protein-coding genes were assigned to the “metabolic process” subcategory (Figure 2). To further enrich our understanding of the functions of the predicted proteins in *P. simonii*, we successfully annotated 11,866 putative proteins in the KEGG database. Detailed KEGG function classifications information is shown in Figure S5. Approximately 87.6% (39,833) of the protein-coding genes were functionally annotated using at least one public databases, including Nr, Swissport, Pfam, KOG, KEGG, and GO (Table S4; File S6). Figure S6 displays the Venn diagram of the protein-coding genes with functional annotations.

### Comparative and evolutionary genomics

**Figure 3:**
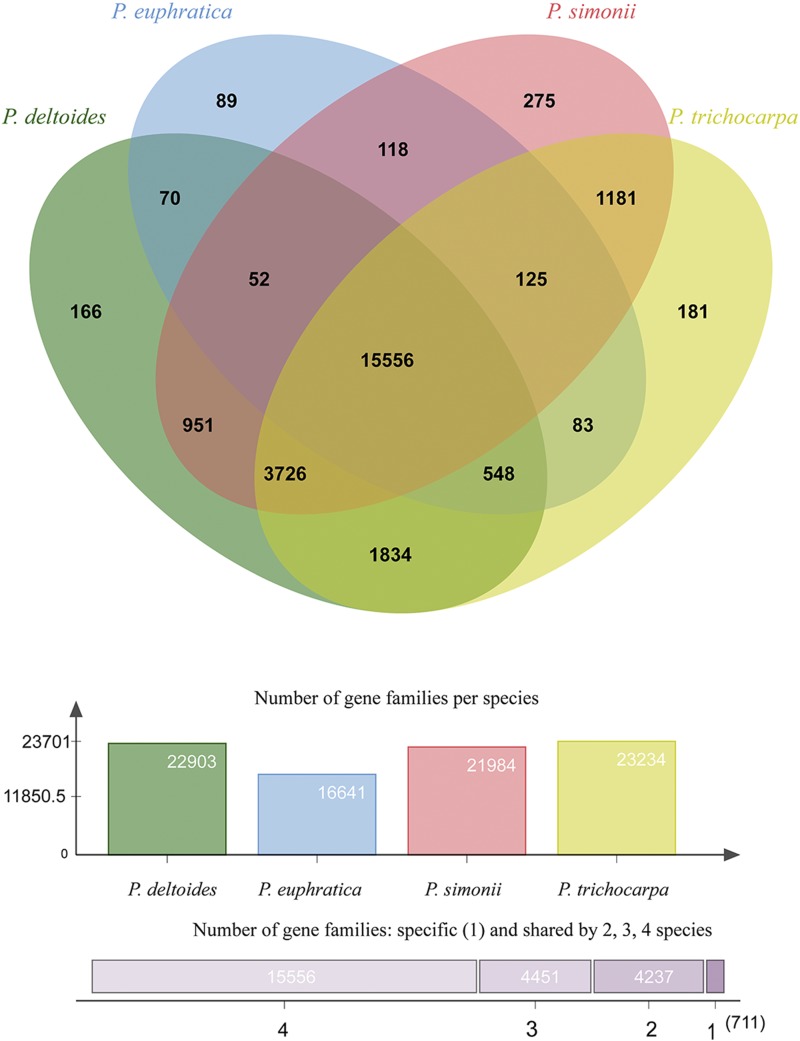
Shared gene families and their distribution per species. Venn diagram showing the shared gene families between the selected *Populus* species: *Populus simonii*, *Populus trichocarpa*, *Populus deltoides*, and *Populus euphratica*. The histogram represents the total number of gene families for each species. The numbers of gene families shared by 4, 3, 2, and only one of these species are presented at the bottom.

Among *P. simonii* and the related eight plant species described in Materials and Methods, a total of 26,181 gene families were constructed using the software OrthoFinder; of these, 966 were identified as single-copy orthologous gene families, and 10 were identified as unique to *P. simonii* (Tables S5 and S6). There were 24,955 gene families containing sequences from the four *Populus* species, of which 15,556 (62.3%), 4,451 (17.8%), 4,237 (17.0%), and 711 (2.8%) were shared by 4, 3, 2, and only one of these species, respectively (Figure 3). Excluding the gene families shared by all four *Populus* species, we found that the desert tree species *P. euphratica* shared 996 gene families with any one or two of the three species *P. trichocarpa*, *P. deltoides*, and *P. simonii*, while 7,692 gene families were shared within the remaining three *Populus* species. The results show that the average number of gene families (2,564) shared within the three *Populus* species was almost 2.6 times the number of gene families shared between *P. euphratica* and any one or two of the other three *Populus* species. This indicates that *P. simonii* is more closely related to *P. trichocarpa* and *P. deltoides* than to *P. euphratica*. In particular, the genome structure of *P. simonii* was evaluated by comparing it with that of *P. trichocarpa*, performed by genomic alignments at the chromosome level using MUMmer software (Kurtz *et al.* 2004). As a result, *P. simonii* structure was globally similar to that of *P. trichocarpa*, without large rearrangements, invertions or translocations (Figure S7).

**Figure 4:**
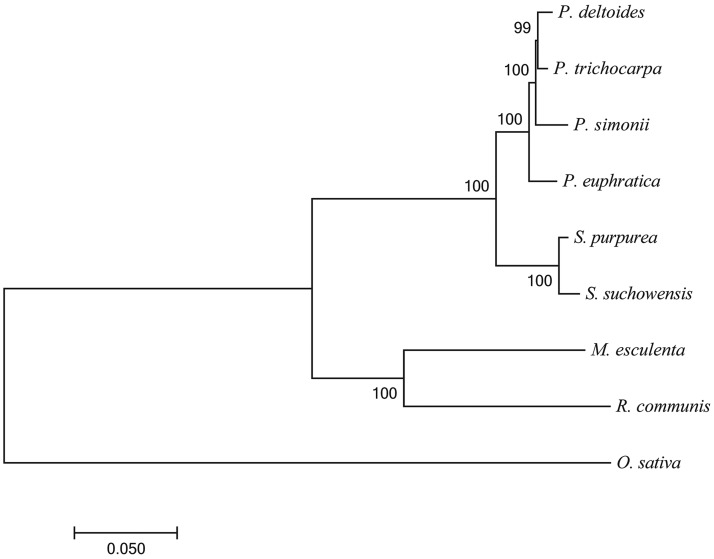
Phylogenetic relationships of *Populus simonii* and related species. A maximum likelihood phylogenetic tree of *P. simonii* and 8 other plant species was constructed through the concatenated alignment of 966 1-to-1 single-copy orthologous genes and then using RAxML software with the model JTT+G+F. The number on the nodes represents the bootstrap support value estimated from 1000 bootstrap tests.

The evolutionary relationship of *P. simonii* to the other eight species was constructed using the 966 single-copy orthologous genes, and the divergence time and the extrapolated calibration time point were estimated. The phylogenetic tree analysis showed that *P. simonii* is more closely related to *P. deltoides* and *P. trichocarpa* than to *P. euphratica* (Figure 4), consistent with the above inference from the number of shared gene families. However, the analysis also showed that *P. trichocarpa* is more closely related to *P. deltoides* than to *P. simonii*, which is contrary to the traditional section classification according to morphology (Eckenwalder 1996) but consistent with the results in recent studies using molecular data (Wang *et al.* 2014; Zong *et al.* 2019); this suggests that *P. simonii* and *P. trichocarpa* should be assigned to different sections in the genus *Populus* (see more in Discussion). Moreover, it was estimated that *P. simonii* diverged approximately 4.36 million years ago from its common ancestor with *P. deltoides* and *P. trichocarpa* (Figure S8). The number of gene duplications was also estimated along each branch on the species tree; this indicated that there were 2,777 gene duplications in *P. simonii* (Figure S9). Furthermore, from the density curves of Ks distributions for the paralogs of *P. simonii* and *P. trichocarpa* (Figure S10), we observed that the two species shared similar peaks near the Ks value of 0.23. This indicated that *P. simonii* experienced the same WGD events as *P. trichocarpa* (Ma *et al.* 2019; Liu *et al.* 2019). Additionally, the analysis of gene family expansion and contraction showed that 2,356 gene families were expanded and 5,224 families were contracted in the *P. simonii* genome compared to the other plant species (Figure S11 and Table S7).

## Discussion

High-quality genome assembly is particularly important for improving the annotation of gene models as well as for facilitating evolutionary and functional genomics analyses (Lin *et al.* 2018). Here, we present a chromosome-scale genome assembly of *P. simonii* with fewer gaps and higher continuity. This assembly was obtained by combining the use of PacBio long reads with the use of Illumina short reads and the related genetic linkage maps. The assembly described here can be considered a high-quality draft genome of *P. simonii* because we attempted to maximize its quality by optimizing the parameters of FALCON, an undertaking that has not previously been reported, as well as by performing two polishing iterations using different sequencing technologies. The size of the assembly was approximately 441 Mb with a contig N50 of 1.94 Mb. Compared with previously published genomes in *Populus*, such as *P. alba* with contig N50 of 9.8 kb (Ma *et al.* 2019) and *P. pruinosa* with contig N50 of 14 kb (Yang *et al.* 2017), the contig N50 of the *P. simonii* genome obtained by SMRT data are greatly improved. Furthermore, the PacBio-based assembly contains more complete repetitive sequences than the Illumina-based assembly (Winkler *et al.* 2018). This confirms that the strategy of combining third-generation sequencing (used to obtain contig with higher continuity) and Illumina sequencing technology (used to obtain high-quality base sequences) can be effectively applied to the assembly of complex plant genomes (Li *et al.* 2017; Jung *et al.* 2019). However, the contig N50 value of our assembly is still far from that of the other plant genomes assembled using the PacBio Sequel platform, such as *Hordeum vulgare* (Zeng *et al.* 2018) and *Brassica rapa* (Zhang *et al.* 2018). This may be due to the higher heterozygosity of the genomes of forest trees and could be improved by increasing the sequencing depth of the PacBio long reads in *P. simonii* from the current ∼50X to 80X (Lin *et al.* 2018).

On the other hand, the quality of a chromosome-level genome assembly largely depends on the genetic linkage map used to anchor the contigs. The key problem in creating a linkage map is whether the order of the molecular markers within each linkage group is sufficiently accurate. Errors in the order of markers in a linkage map will seriously affect the order of contigs in the anchoring process. We used two high-density genetic linkage maps to anchor 336 contigs into chromosomes with a size of 398 Mb, accounting for 90.2% of the genome in this study. The linkage maps were constructed using SNPs generated from the RADseq data across the mapping population in our previous study (Yao *et al.* 2018). Compared with previous genetic mapping studies in *Populus* (Bradshaw *et al.* 1994; Yin *et al.* 2002; Zhang *et al.* 2004; Zhang *et al.* 2009; Paolucci *et al.* 2010), the linkage maps used in this study could be considered to be of high quality because they contained many more SNPs that were confirmed to be of high quality. Moreover, the number of divided linkage groups perfectly matched the karyotype of *Populus* under a wide range of LOD thresholds, which was rarely reported in the previous studies. This suggests that the SNPs were uniformly distributed on the genome and were of high quality. Undoubtedly, high-quality SNPs can improve the accuracy of ordering SNPs using the available mapping software, thus enhancing the quality of our chromosome-level genome assembly. However, there is still room to improve the algorithms used to order markers in the current mapping software; ordering tens or hundreds of markers in a linkage group belongs to the NP-hard category of problems (Wu *et al.* 2008; Monroe *et al.* 2017), which involves choosing the optimal order of the markers among a huge number of possible orders. Advances in ordering markers would substantially increase the precision of genetic mapping and hence improve the accuracy of chromosome-level genome assembly.

In the context of gene predictions and annotations, our assembled genome has some characteristics similar to those of previous genome assembly studies in *Populus*. The annotation of the *P. simonii* genome is almost complete, with 97.9% of the complete BUSCOs, higher than the completeness of annotation in *P. alba* (91.10%) (Ma *et al.* 2019), *P. trichocarpa* (93.95%) (Tuskan *et al.* 2006) and *P. euphratica* (94.35%) (Ma *et al.* 2013). Approximately 41.47% of the genome was annotated as containing repetitive sequences, similar to the values estimated for other *Populus species* (Tuskan *et al.* 2006; Ma *et al.* 2013; Yang *et al.* 2017; Ma *et al.* 2019). A total of 45,459 genes were predicted in the genome of *P. simonii*, slightly more than the number of genes predicted in *P. trichocarpa* (42,950 genes) and in *P. deltoides* (44,853 genes). The expansion in the number of predicted genes may be attributed to repeated gene duplication (Yang *et al.* 2018); this could be confirmed by analyzing the whole-genome duplications as well as the gene families. Among the predicted genes, 39,833 (87.6%) were annotated with relevant functional information, laying the foundation for further elucidation of the biological characteristics of *P. simonii*, such as its drought stress response, its barren tolerance and its developed root system. Overall, the acquisition of the genome of *P. simonii* provides an important genetic resource for comparative genomics in *Populus* and facilitates further studies on phylogenetic inference and whole-genome duplication events.

We measured the phylogenetic affinity of *P. simonii* with three other *Populus* species, *P. deltoides*, *P. trichocarpa*, and *P. euphratica*, because these species provide the only genome data available to date in *Populus*. According to important morphological characteristics, Eckenwalder (1996) assigned *P. simonii* and *P. trichocarpa* to the section *Tacamahaca*, *P. deltoides* to the section *Aigeiros*, and *P. euphratica* to the section *Turanga*, suggesting that *P. simonii* and *P. trichocarpa* are more closely related than any other two of the four species. This classification of the *Populus* species is not consistent with our current phylogenetic result, which shows that *P. deltoides* and *P. trichocarpa* are more closely related to each other than *P. simonii* is to either *P. trichocarpa* or *P. deltoides*. Interestingly, this is not the first report of an inconsistent phylogenetic relationship within the *Populus* genus based on the use of different methods. Using nuclear and plastid DNA sequences, Wang *et al.* (2014) found that the section *Tacamahaca* could be divided into two groups, with *P. simonii* belonging to section *Leucoides* or to a sister group to section *Aigeriros* and *P. trichocarpa* belonging to the other group as a subsection in *Tacamahaca*. More recently, Zong *et al.* (2019) divided *P. simonii* and *P. trichocarpa* into different clades but placed *P. deltoides* and *P. trichocarpa* in the same clade using whole chloroplast genome sequences. Overall, in addition to the fact that there is almost no controversy regarding the relationship of *P. euphratica* to other *Populus* species, the phylogenetic relationships among *P. simonii*, *P. deltoides*, and *P. trichocarpa* revealed by these two studies are consistent with the results obtained in the current study.

## Conclusions

Herein, we report the first chromosome-scale genome assembly for *P. simonii*; it was obtained through an assembly strategy that combined the use of PacBio long reads data with the use of Illumina short PE reads data and related genetic linkage maps. With the assembly data, phylogenetic analysis indicates that *P. simonii* and *P. trichocarpa* should be assigned to different sections in the genus *Populus*. The genome assembly, which includes predicted genes and functional annotations, not only provides an important resource for the comparative and functional genomics of *Populus* species, but also provides the closest reference sequences for identifying genomic variants in an F_1_ hybrid population derived by crossing *P. simonii* and other *Populus* species. The assembly strategy used in this work can be applied to other species in *Populus* to generate chromosome-level genome assemblies in a fast and cost-effective manner.
